# Hepatitis C virus in healthy blood donors in Sri Lanka

**DOI:** 10.4103/0973-6247.75976

**Published:** 2011-01

**Authors:** Dammika Senevirathna, Senani Amuduwage, Shirani Weerasingam, Saroj Jayasinghe, Neil Fernandopulle

**Affiliations:** *Genetech Molecular Diagnostics and School of Gene Technology, University of Colombo, Colombo, Sri Lanka*; 1 *National Blood Transfusion Center, University of Colombo, Colombo, Sri Lanka*; 2 *Faculty of Medicine, University of Colombo, Colombo, Sri Lanka*

**Keywords:** Blood donors, Hepacivirus, genotype

## Abstract

**Introduction:**

Hepatitis C virus (HCV) is the etiological agent for the majority of cases of non-A, non-B hepatitis. As a blood-borne virus, HCV is widely recognized as a major causative agent of post-transfusion non-A, non-B hepatitis. The prevalence of HCV and the distribution of HCV genotypes in Sri Lanka in comparison with the rest of Asia are not well known.

**Materials and Methods:**

The blood samples collected from healthy blood donors at the National Blood Transfusion Centre of Sri Lanka were screened to determine the prevalence and the genotypes of HCV among blood donors in Sri Lanka.

**Results:**

HCV antibodies were found in 53 of 4980 blood donors. However, of the 53 only 8 positive results were confirmed by Reverse Transcription-PCR, which suggests frequent false-positive results or viral clearance. The PCR positive samples were genotyped by DNA sequencing of the Core/E1 regions of HCV genome, and all the HCV viruses belonged to genotype 3, of which 7 were 3a and 1 was 3b.

**Conclusion:**

HCV is relatively rare among blood donors in Sri Lanka and only genotype 3 was detected in the studied group.

## Introduction

Hepatitis C virus (HCV), first identified in 1989, causes a slowly progressive disease affecting about 170 million (3%) people worldwide.[1,2] More than three million new cases of infection are reported annually, and epidemiological studies indicate a wide variation in its prevalence patterns in different continents and countries.[2] Sri Lanka lacks data on the prevalence of HCV in the general population as well as in healthy blood donors, but there have been a few studies reporting the seroprevalance of HCV antibodies among the patients with alcoholic cirrhosis[3] and patients who have had multiple transfusions.[4]

The genome of HCV is a single-stranded, positive-sense RNA molecule of approximately 9.6 kb in length.[1] There is a remarkable genetic heterogeneity and divergence among HCV sequences which has lead to the categorization of HCV into “genotypes”. HCV genotypes are related to regional distribution,[5] clinical manifestation, response to treatment, and prognosis of HCV infection.[6]

Therefore, the study was designed to fulfill two objectives. The first was to determine the prevalence of HCV among blood donors in Sri Lanka by testing specimens for HCV antibodies and RNA. The second was to genotype HCV RNA-positive specimens and to determine the phylogenetic relationship between strains by means of DNA sequence analysis.

## Materials and Methods

A total of 4980 blood samples (representing all districts in Sri Lanka) were collected from blood donors who donated blood to the National Blood Transfusion Centre, Colombo, Sri Lanka at their first donation between August and December 2009. All the samples were tested for HCV antibodies. Antibody positive samples were tested for HCV RNA, and the RNA positive samples were genotyped by DNA sequence analysis.

Serum samples were tested by using an enzyme immuno assay (EIA) for HCV antibodies to recombinant antigens Core, NS3, NS4, and NS5 (INNOTEST HCV Ab IV, Innogenetics, Belgium) according to the manufacturer’s instructions. The samples which showed a wide range of antibody titer ranging from “marginally positive” to “strongly positive” were taken as seropositives for this study. The repeat reactivity for HCV antibodies was not tested. Guanidium thiocyanate/silica RNA extraction was carried out as previously described by Boom *et al.*,[7] and HCV RNA was detected by Reverse Transcription-Polymerase Chain Reaction (RT-PCR) using primers derived from the highly conserved the 5’ untranslated (5’-UTR) genomic region as previously described.[8]

HCV RNA-positive specimens were further characterized by sequencing parts of the Core/E1 and NS5B regions. Briefly, the purified RNA was used to generate cDNA by reverse transcription. Nested PCR was performed with sets of published primers to amplify DNA from Core/E1 or NS5B regions.[9] The amplified products were separated in an agarose gel and purified with the Promega Wizard® PCR preps DNA purification system (Promega, Madison, WI, USA). DNA sequencing was performed at Eton BioScience, USA. The sequences were aligned in the BioEdit sequence alignment editor version 7.0.9.0[10] by using the Clustal W Multiple alignment.[11] Phylogenetic trees for HCV which were based on Core/E1 and NS5B sequences and genetic distances were calculated with MEGA software version 4[12] using the Maximum Likelihood model. The sequences of Core/E1 and NS5B of HCV strains in Sri Lanka were deposited in NCBI GenBank under the accession numbers given in Table 1.

**Table 1:** Subtype and GenBank accession numbers of HCVs in this study

Specimen	Core/E1		NS5B	
	Accession no.	Subtype	Accession no.	Subtype
SLHC15	EU849145	3a	EU867442	3a
SLHC16	*	*	EU867443	3a
SLHC17	FJ236901	3a	FJ236905	3a
SLHC18	FJ236902	3a	FJ236906	3a
SLHC19	FJ236903	3a	FJ236907	3a
SLHC20	GU075872	3a	GU075878	3a
SLHC21	GU075873	3a	GU075877	3a
SLHC24	FJ236904	3b	*	*

* Could not be sequenced successfully.

## Results

Of 4980 blood donors, only 53 (1.06%) were positive for anti-HCV antibodies and of that, 8 (15.09%) were positive for HCV RNA by RT-PCR. Of the eight isolates, seven belonged to HCV subtype 3a and one belonged to subtype 3b. The type 3a isolates had a mean genetic distance of 0.102.

## Discussion

The goal of this study was to determine the prevalence of HCV infection and to determine the genotypes of HCV in a cohort of blood donors in Sri Lanka. We screened 4980 samples from the National Blood Transfusion Centre of Sri Lanka for HCV antibodies and then tested antibody-positive samples by RT-PCR to confirm the HCV infection. Only 1.06% of the blood donors were positive for HCV antibodies and if we assume that seronegative donors were not viremic, only 0.16% of the blood donors were positive for HCV RNA indicating that HCV is rare among blood donors in Sri Lanka. Two probable reasons for this are that the Sri Lanka National Blood Transfusion Centre screens all blood donors for HCV prior to transfusion and low numbers of injecting drug users in Sri Lanka.[13]

We detected 53 HCV antibody positive specimens, but only 8 (15.09%) of these were confirmed by RT-PCR. These results may indicate that false-positive ELISA results for HCV are frequent. False-positive results could be due to the nonspecific antibody binding or to cross-reactivity with other circulating organisms.[14] Alternatively, perhaps true antibody positive participants did not have positive RT-PCR results because they had cleared HCV viremia.[14]

**Figure 1:**
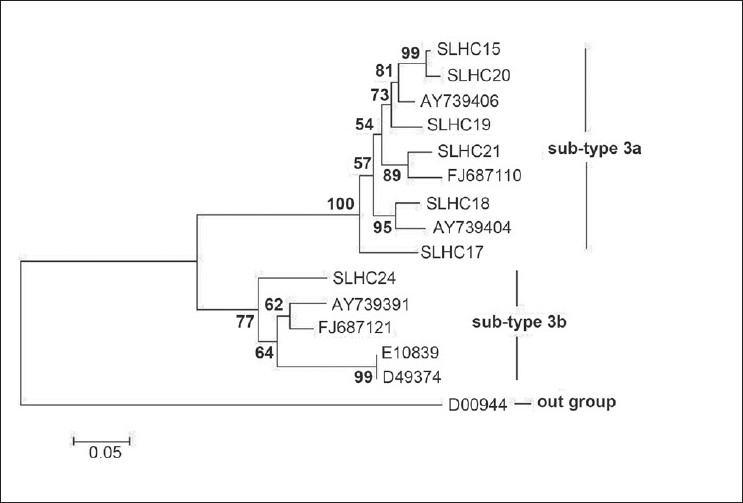
A phylogenetic tree based on the Core/E1 region. The specimen from this study is labelled with the initials “SLHC” and their accession numbers were given in Table 1. Reference sequences used are labelled with their accession numbers

HCV is genetically heterogeneous, and strains can be grouped into six major genotypes that have distinct geographical distributions.[15] We were able to sequence the Core/E1 and NS5B regions of eight RNA-positive samples. One Core/E1 and one NS5B (from different samples) were excluded from further analysis because of nonsense substitutions. To determine the genotype and subtype of Sri Lankan HCVs, we created a phylogenetic tree using Core/E1 sequences and genotype 1–6 reference sequences obtained from NCBI GenBank [Figure 1]. We only detected genotype 3 in these specimens from healthy blood donors. Seven samples belonged to subtype 3a and the remaining one to subtype 3b. To our knowledge this is the first report of HCV genotypes in blood donors in Sri Lanka. Genetic distances between isolates of a virus could give an indication of the epidemiology of that infection. Low genetic distance between isolates is indicative of recent spreading of the virus from local sources, whereas greater genetic distances indicate either regionalized spreading within a country or the introduction of isolates from external sources.[16] Genetic distance measurements among the Sri Lankan genotype 3a Core/E1 region was compared with a group of Core/E1 sequences from a study carried out in Pakistan, where genotype 3 is the most prevalent and a high rate of internal transmission has been recorded.[17] While the Pakistani sequences were closely related to each other (the mean genetic distance was 0.031), the Sri Lankan genotype 3a isolates displayed much greater genetic distance within the group (the mean genetic distance was 0.102), perhaps indicating a low level of recent localized transmission.

The blood transfusion services in Sri Lanka use the EIA tests mainly for the purpose of screening the donated blood and discarding any samples that are seropositive. However, because of the high proportion of false-positive results in the EIA tests, the donors are not notified of their seropositive status as no confirmatory test is performed on the seropositive samples. However, performing confirmatory assays on all seropositive donors would help to immediately detect and treat HCV-positive donors. This would also help to reduce the spread of HCV in the country.
